# Predictive Factors for Surgical Intervention in Neonates with Necrotizing Enterocolitis: A Retrospective Study

**DOI:** 10.3389/fsurg.2022.889321

**Published:** 2022-05-17

**Authors:** Lei Yu, Chao Liu, Qingjing Du, Lishuang Ma

**Affiliations:** ^1^ Children's Hospital, Capital Institute of Pediatrics, Department of Hospital Infection Administrative, Beijing, China; ^2^ Children's Hospital, Capital Institute of Pediatrics, Department of Neonatal General Surgery, Beijing, China

**Keywords:** laboratory examination, necrotizing enterocolitis, operation time, physical examination, retrospective study

## Abstract

**Background:**

The current indications based on the clinicopathological parameters for predicting the need for surgery in neonatal necrotizing enterocolitis (NEC) are still limited. This study retrospectively analyzes the characteristics of neonatal NEC and aims to identify the risk factors for surgical intervention in NEC.

**Methods:**

Data of the NEC cases from 2015 to 2019 were collected from our institution and divided into two groups: surgical group (*n* = 41) and conservative treatment group (*n* = 143). Clinical, physical, and laboratory measures were analyzed by univariate analysis and multivariate logistic regression. The diagnostic values and receiver operative characteristic (ROC) curve were used for the assessment.

**Results:**

Univariate analysis identified significant differences between the surgical group and the conservative group in a series of clinical, physical, and laboratory measures (all *p* < 0.05). The results of multivariate logistic regression analysis showed that procalcitonin (adjusted OR: 167.1, 95% CI, 3.585–7,788.758, *p* = 0.009) and gestational age (adjusted OR: 0.85, 95% CI, 0.77–0.94, *p* = 0.001) were independent surgical indications for NEC. The results from ROC curve and diagnosis values demonstrated that procalcitonin [the area under the curve (AUC) = 0.864], CRP (AUC = 0.783) and fibrinogen (AUC = 0.720) had good predictive performance for surgical NEC.

**Conclusions:**

The level of procalcitonin and gestational age were found to be independent surgical indications for neonates with NEC.

## Introduction

Necrotizing enterocolitis (NEC), a gastrointestinal tract disease, is the most common gastrointestinal emergency among neonates, which is associated with high morbidity and mortality (1–3). The frequency of mortality resulting from NEC ranges from 20% to 30%, the highest in the smallest infants and those requiring surgery (4–6). Most cases of NEC are initially managed conservatively with optimum medical treatment, ventilatory support, parenteral nutrition, abdominal decompression, and antibiotics. In 27%–52% of the infants with NEC, a surgical intervention including primary peritoneal drains or laparotomy with bowel resection and the creation of an ileostomy is indicated during its disease course (7, 8), and without definitive indications for surgical intervention, the care of infants with NEC has been a contentious issue for pediatric surgeons.

The current and absolute indication for surgery in NEC patients is evidence of perforation on the abdominal radiograph (4), but more than 50% of infants who receive surgery for NEC do not show any sign of perforation (9). To date, studies investigating the clinically relevant predictive factors and serum markers for surgical intervention are still limited (10, 11). A survey of practice in the United Kingdom of pediatric surgeons found a lack of agreement in the absolute and relative indications for surgical intervention in NEC patients (12). In addition, radiographic evidence of pneumoperitoneum remains the only absolute criteria for peritoneal drain or laparotomy in NEC, but there is no consensus on the utility of physical exam findings to assist in differentiating stable medical NEC from that which ultimately requires surgical intervention (13). An identification of the predictors of surgical needs is important, since this may facilitate early decisive management such as surgical consultation, additional diagnostics (laboratory investigations or radiographic imaging), transportation to a surgical center, and surgical intervention (14). A comprehensive multivariate analysis may help to identify the risk factors for patients who do not display any evidence of perforation but who do need surgical intervention.

It is under such circumstances that we conducted this retrospective study to identify the clinical characteristics from physical exam and laboratory parameters that might potentially predict the needs of surgical intervention for deteriorated NEC in preterm infants. An identification of these factors may assist with the resolution of surgical intervention before the developed stage of NEC in this patient population.

## Materials and Methods

### Patients

This single-centered retrospective case-control study included data of newborn patients in the Children’s Hospital of Capital Institute of Pediatrics from January 2015 to December 2019. The electronic medical records for demographic information, clinical management, and laboratory data were reviewed. The study was approved by the Ethics Committee of Capital Institute of Pediatrics (SHERLLM2022001) and was performed in accordance with the Declaration of Helsinki. The need for written informed consent from their legal guardians was waived by the Ethics Committee of Capital Institute of Pediatrics because this was a retrospective study, and the data were collected and interpreted anonymously. The inclusion criteria were as follows: (1) newborn patients were diagnosed with NEC by the 4th Edition of *Practice of Neonatology*; (2) medical records, including demographic and clinical information, were complete. Patients were excluded if they had serious complications, sepsis, or died within 48 h after admission. Those who had intestinal surgery after NEC-related treatment were also excluded from this study.

### Grouping

The eligible patients were divided into either of the two groups, surgical group or conservative group, based on their condition during hospitalization. According to a wildly recognized consensus on the choice of treatments for neonates with NEC in China, the main surgical indications are the presence of pneumoperitoneum and intestinal necrosis or whether bile or stool can be seen in the abdominal puncture fluid. For those without the symptoms of intestinal perforation, the presence of persistent abdominal distension, non-exudative ascites, abdominal wall erythema, palpable abdominal mass, and hypotension after conservative treatment was also the surgical exploration indication. All patients in our study received conservative treatment, including water restriction, gastrointestinal decompression, the use of broad-spectrum antibiotics, intravenous nutritional support, and symptomatic treatment after admission, and those who presented with poor efficacy proceeded to surgical intervention. Finally, there were 41 patients, including 23, who had intestinal perforation included in the surgical group.

### Data Collection

Data in the surgical group were obtained upon the latest examination before surgery, and the data in the conservative group were collected upon admission. Demographic data including gender, age (day), and weight when diagnosis with NEC were included in this analysis. Clinical information including the presence of fever, emesis, gross bloody stool, apnea, pneumoperitoneum, rigid muscle, weak bowel sound, and gastric retention were also retrieved from the medical records. Laboratory examination was performed after the diagnosis of NEC. The levels of white blood cell (WBC), platelet count (PLT), neutrophils, eosinophil, C-reactive protein (CRP), procalcitonin (PCT), blood PH, lactate, fibrinogen, prealbumin (PA), albumin (Alb), glucose, and serum sodium were measured.

### Statistical Analysis

The normality of data distribution was tested by using the Kolmogorov–Smirnov test. Continuous variables were expressed as means ± standard deviation or medians (IQR), and the group differences were assessed by using a *t*-test or Mann–Whitney *U* tests as appropriate for the data distribution. Categorical variables were expressed as the number of cases or the percentages (%). Chi-squared or Fisher’s exact tests were adopted for assessing the differences between groups. Univariate and multivariable logistic regression was performed to identify independent clinical predictors for implementing surgical intervention in patients with NEC. Stepwise selection was applied to build the final model. And, an odds ratio (OR) with a 95% confidence interval (CI) was calculated. The diagnostic value was analyzed by using the receiver operating characteristic (ROC) curve to calculate the best cut-off, as well as by using the area under the curve (AUC), Youden index, sensitivity, and specificity. A *p* value of less than 0.05 was considered significant. All analyses were performed using IBM SPSS Statistics v21 (IBM Corporation, NY, USA).

## Results

### Patients’ Characteristics

During the study period, the records of 184 eligible newborn patients with NEC were included in this analysis. Of these, 41 patients underwent surgical intervention and 143 patients received conservative treatment. The demographic and clinical characteristics of the patients are listed in Table 1. The gestation age (*p* = 0.001) and weight at diagnosis (*p* = 0.002) between the surgical group and the conservative group were significantly different. The gender and age at diagnosis were not different between the two groups (all *p* > 0.05).

**Table 1:** Patients’ characteristics between surgical group and conservative group.

	Surgical group (*N* = 41)	Conservative group (*N* = 143)	*p*
Gender, *N* (%)			0.592
Boy	21 (51.22)	80 (55.94)	
Girl	20 (48.78)	63 (44.06)	
Gestational age, *N* (%)			0.001
<32 weeks	13 (31.71)	19 (13.29)	
32–36 weeks	19 (46.34)	48 (33.57)	
≥37 weeks	9 (21.95)	76 (53.15)	
Weight at diagnosis, *N* (%)			0.002
<1,500 g	7 (17.07)	12 (8.39)	
1,500–2,500 g	24 (58.54)	51 (35.66)	
>2,500 g	10 (24.39)	80 (55.94)	
Age at diagnosis, *N* (%)			0.953
<7 days	15 (36.59)	26 (18.18)	
7–13 days	11 (26.83)	36 (25.17)	
≥14 days	15 (36.59)	51 (35.66)	
Clinical information			
Fever, *N* (%)	9 (21.95)	13 (9.09)	0.025
Poor mental reaction, *N* (%)	24 (58.54)	37 (25.87)	<0.001
Emesis, *N* (%)	10 (24.39)	38 (26.57)	0.779
Gross bloody stool, *N* (%)	20 (48.78)	84 (58.74)	0.257
Apnea, *N* (%)	4 (9.76)	12 (8.39)	0.750
Abdominal distension, *N* (%)	41 (100)	81 (56.64)	<0.001
Rigid muscle, *N* (%)	24 (58.54)	18 (12.59)	<0.001
Weak bowel sound, *N* (%)	34 (82.93)	47 (32.87)	<0.001
Gastric retention, *N* (%)	5 (12.20)	17 (11.89)	0.957
Colored beverage on gastric returns after gastric tube replacement, *N* (%)	7 (4.90)	13 (9.09)	0.148
Bell’s stage, *N* (%)			<0.0001
II	0 (0)	139 (97.2)	
IIIA	18 (43.9)	3 (2.1)	
IIIB	23 (56.1)	1 (0.7)	
Depth of bacterial invasion, *N* (%)			<0.0001
Moderate	24 (58.5)	8 (5.6)	
Severity	17 (41.5)	135 (94.4)	

The clinical responses of the newborn patients were compared between the surgical group and the conservative group. The results presented that patients from the surgical group had a higher percentage of fever (*p* = 0.025), poor mental reaction (*p* < 0.001), pneumoperitoneum (*p* < 0.001), rigid muscle (*p* < 0.001), weak bowel sound (*p* < 0.001), a high stage of Bell’s stage (*p* < 0.001), and a high rate of moderate depth of bacterial invasion than the conservative group. But no significant difference was found in the presence of emesis, gross bloody stool, apnea, gastric retention, and colored beverage on gastric returns after gastric tube replacement between the two groups.

### Laboratory Tests

The comparisons of laboratory variables showed that the levels of WBC (8.47 ± 5.59 vs. 11.53 ± 4.71), platelet count (224.29 ± 167.74 vs. 229.69 ± 129.94), eosinophil (0.08 ± 0.19 vs. 0.48 ± 0.60), blood pH (7.33 ± 0.10 vs. 7.39 ± 0.98), albumin (28.132 ± 6.041 vs. 31.270 ± 3.496), and serum sodium (137.76 ± 4.75 vs. 139.64 ± 3.68) were significantly lower in the surgical group than in the conservative group (all *p* < 0.05, Table 2). And the levels of CRP (47.92 ± 46.17 vs. 10.43 ± 23.14), PCT (27.17 ± 48.97 vs. 1.36 ± 4.02), lactate (3.19 ± 2.77 vs. 2.15 ± 2.21), and fibrinogen (2.769 ± 1.195 vs. 2.172 ± 0.839) were significantly higher in the surgical group than in the conservative group (All *p *< 0.05).

**Table 2:** Comparisons of laboratory variables between the surgical group and the conservative group.

	Surgical group (*N* = 41)	Conservative group (*N* = 143)	*p*
WBC (10^9^/L)	8.47 ± 5.59	11.53 ± 4.71	0.001
Neutrophils (10^9^/L)	5.23 ± 3.97	5.28 ± 3.31	0.937
Platelet count (10^9^/L)	224.29 ± 167.74	229.69 ± 129.94	0.003
Eosinophil (10^9^/L)	0.08 ± 0.19	0.48 ± 0.60	<0.001
CRP (mg/L)	47.92 ± 46.17	10.43 ± 23.14	<0.001
PCT (ng/mL)	27.17 ± 48.97	1.36 ± 4.02	<0.001
Blood pH	7.33 ± 0.10	7.39 ± 0.98	0.002
Lactate (mmol/L)	3.19 ± 2.77	2.15 ± 2.21	0.024
Fibrinogen (g/L)	2.769 ± 1.195	2.172 ± 0.839	0.001
Albumin (g/L)	28.132 ± 6.041	31.270 ± 3.496	<0.001
Blood glucose (mmol/L)	4.860 ± 2.621	4.313 ± 1.785	0.126
Prealbumin (g/L)	83.27 ± 21.60	86.27 ± 27.89	0.680
Serum sodium (mmol/L)	137.76 ± 4.75	139.64 ± 3.68	0.008

*WBC, white blood cell; CRP, C-reactive protein; PCT, procalcitonin.*

### Multivariate Logistics Regression

Univariate analysis identified 17 potential risk factors, namely, gestational age, weight at diagnosis, poor mental reaction, fever, apnea, rigid muscle, weak bowel sound, WBC, platelet count, eosinophil, CRP, PCT, blood pH, lactate, fibrinogen, albumin, and serum sodium for implementing surgical treatment in NEC patients (all *p* < 0.05, results not given). We used the stepwise selection in multivariate logistic regression to build the final model. The results showed that only the gestational age (OR: 0.217, 95% CI, 0.056–0.844, *p* = 0.028) and the PCT level (OR: 167.1, 95% CI, 3.585–7,788.758, *p* = 0.009) were the independent risk factors for implementing surgical treatment (Table 3).

**Table 3:** Multivariate logistic regression for implementing surgical treatment in NEC patients.

	OR	95% CI	*p*
PCT	167.1	3.585, 7,788.758	0.009
Gestational age (weeks)	0.217	0.056, 0.844	0.028

*PCT, procalcitonin.*

### Diagnostic Performance of the Laboratory Variables

**Figure 1:**
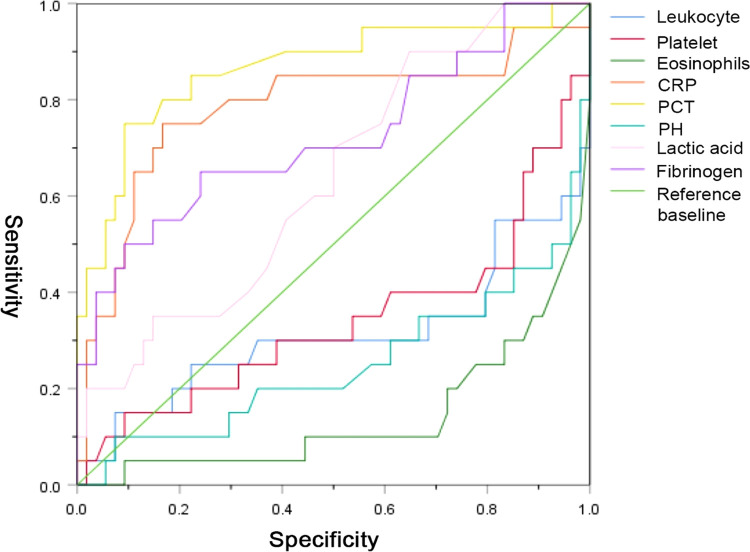
The diagnostic values of the laboratory variables for implementing surgical treatment in patients with necrotizing enterocolitis NEC.

We used the ROC curve to identify the diagnostic performance of the laboratory variables for implementing surgical treatment in NEC. The results in Figure 1 show that PCT, CRP, fibrinogen, and lactate might be able to predict the need for surgery among NEC patients. We further assessed their diagnostic values, and the results demonstrated that PCT (AUC = 0.864) had a promising diagnostic performance under the cut-off point of 1.825 ng/ml. Detailed information of the diagnostic value is presented in Table 4.

**Table 4:** Diagnostic characteristics of CRP, PCT, lactic acids, and fibrinogen for implementing surgical treatment in NEC patients.

	AUC	Sensitivity	Specificity	Youden index	Cut-off point
CRP (mg/L)	0.783	0.75	0.833	0.583	18.5
PCT (ng/ml)	0.864	0.75	0.907	0.657	1.825
Lactate (mmol/L)	0.636	0.90	0.352	0.252	1.250
Fibrinogen (g/L)	0.720	0.65	0.759	0.409	2.495

*CRP, C-reactive protein; PCT, procalcitonin; AUC, area under the curve; NEC, necrotizing enterocolitis.*

## Discussion

In this study, we identified that PCT and gestational age were independent surgical indications for neonates with NEC. In the univariate analysis, we found a series of clinical characteristics from physical exam and laboratory parameters including pneumoperitoneum, weak bowel sound, gastric retention, platelet count, and CRP associated with the need for surgical intervention, but the adjusted OR indicated no significant association.

Gestational age as an important maternal factor has been recognized in previous studies to be an independent risk factor for both NEC and surgical needs for NEC (15–17). A previous study investigating the association between gestational age and surgical timing and outcomes for NEC observed that an increase in gestation at birth per week might shorten an average of 0.45 h from a suspicion of NEC to first surgical intervention (18). They also found that a lower gestational age is an independent risk factor for increased 28-day mortality or parenteral nutrition requirement (adjusted OR: 0.85, 95% CI, 0.77–0.94, *p* = 0.001). In addition, a recent published multicenter study demonstrated an adjusted OR of 0.91 (95% CI, 0.86–0.96, *p* = 0.001) in gestational age for the need for surgical intervention (19). This inverse association is in accordance with the result in our study, despite the fact that they included birth weight and maternal corticosteroid in their multivariate logistic model, but only PCT was included in our final model.

Previous studies showed that the magnitude of systemic inflammation including an elevated CRP and plasma lactate level might correlate with the severity of NEC and predict unfavorable outcomes (20–22). An international survey among pediatric surgeons reported that the commonly used laboratory variables for predicting surgical NEC are PLT (99%), CRP concentration (90%), WBC (83%), and lactate levels (43%) (23). Yu et al. retrospectively investigated 84 preterm patients with NEC and reported that NEC patients who needed surgery had significantly lower plasma concentrations of WBC and PLT and a higher concentration of CRP than conservatively treated neonates (24). This difference is congruent with the univariate analysis of our study. In addition, we found lower levels of eosinophil, blood pH, albumin, and serum sodium and higher levels of PCT, lactate, and fibrinogen in patients who received surgical treatment. However, of all the laboratory variables, only the level of PCT (adjusted OR: 167.1, 95% CI, 3.585–7,788.758, *p* = 0.009) was considered a significant indication for surgical NEC in our analysis. We suggest that this is reasonable given that PCT is similar in reflecting the intestinal inflammation as CRP and WBC, and the predictive value of PCT has been well demonstrated for the occurrence of post-NEC strictures (25).

Clinical and physical deterioration such as abdominal distention and abdominal wall erythema with tenderness despite optimal conservative therapy is considered a relative indication for surgery in patients with NEC. Tepas et al. observed trends in seven different components of metabolic derangement associated with NEC, including positive blood cultures, acidosis, bandemia, neutropenia, hyponatremia, hypotension, and thrombocytopenia (26). They concluded that the presence of three or more of the components was significantly associated with the need for surgical intervention in NEC. A recent published study using a compositive score based on patients’ physical status assessed the need for surgery in neonates with NEC (15). They found a significant association between the composite physical examination score and the need for surgery with promising sensitivity (0.88) and specificity (0.80). Although the compositive score showed a favorable predictive value in determining the need for surgery, they also found that no single component alone could predict the need for surgery. Similarly, patients who underwent surgical NEC presented with significantly higher chances of fever, poor mental reaction, abdominal distension, rigid muscle, and weak bowel sound compared with the conservative group in our study. But no single component from physical examination was concluded as the independent surgical indication in NEC. In such a situation, it will be necessary to develop a more comprehensive comprised score from physical examination in future studies.

We recognized the limitations of this study. First, this is a single-center case-control study with a relatively small sample size, and an imperfect matching of patients does not allow us to identify predictors but rather correlation between measures and outcome. Second, although we analyzed a large number of risk factors, including clinical and physical characteristics and laboratory parameters in predicting the need for surgery for NEC; however, only PCT and gestational age were finally left in the multivariate model. Additionally, in this study, we did not test the fecal calprotectin, which is a very popular biomarker of intestinal infection.

In conclusion, this study suggested that despite significant differences being observed in a series of clinical, physical, and laboratory parameters based on the univariate comparison between the surgical group and the conservative group, only PCT and gestational age were found to be independent surgical indications for neonates with NEC.

## Contribution to the Field Statement

NEC, a gastrointestinal tract disease, is the most common gastrointestinal emergency among neonates, which is associated with high morbidity and mortality. Most cases of NEC are initially managed conservatively with optimum medical treatment, ventilatory support, parenteral nutrition, abdominal decompression, and antibiotics. In 27%–52% of the infants with NEC, a surgical intervention is indicated, and without definitive indications for surgical intervention, the care of infants with NEC has been a contentious issue for pediatric surgeons. This retrospective study was based on the data of the NEC cases from 2015 to 2019 and aimed to identify the clinical characteristics from physical exam and laboratory parameters. Although only a univariate comparison was performed between the surgery group and the conservative group, significant differences were still observed in a series of clinical, physical, and laboratory parameters. This study suggested that the level of PCT and gestational age were independent surgical indications for neonates with NEC, which may assist with the resolution of surgical intervention before the developed stage of NEC in this patient population.

## Data Availability

The raw data supporting the conclusions of this article will be made available by the authors, without undue reservation.
